# Surgical Outcomes in Patients with Preoperative GLP-1 Therapy: A Retrospective Analysis

**DOI:** 10.1007/s11695-025-08136-5

**Published:** 2025-08-12

**Authors:** Adisa Poljo, Jakob J. Reichl, Simon Bürgi, Amanda S. Dirnberger, Romano Schneider, Jennifer M. Klasen, Adrian T. Billeter, Beat P. Müller, Ralph Peterli, Marko Kraljević

**Affiliations:** 1 Department of Visceral Surgery, Clarunis, University Digestive Health Care Center Basel, St. Clara Hospital and University Hospital Basel, Basel, Switzerland; 2 Department of Cardiology and Cardiovascular Research Institute Basel (CRIB), University Hospital Basel, University of Basel, Basel, Switzerland; 3 Faculty of Medicine, University of Basel, Basel, Switzerland; 4 Department of Clinical Research, University Hospital Basel, Basel, Switzerland

**Keywords:** GLP-1 agonists, Weight loss, Metabolic bariatric surgery, Preoperative treatment

## Abstract

**Background:**

Obesity, a chronic disease with serious associated medical problems, remains a global health challenge. While metabolic bariatric surgery (MBS) is the most effective treatment for weight loss, GLP-1 receptor agonists (RAs) are emerging as promising alternatives or adjuncts. This study evaluates the impact of preoperative GLP-1 therapy on weight loss and obesity-related complications over three years and explores reasons for transitioning to surgery.

**Methods:**

Patients who underwent RYGB or SG from 2015–2021 were retrospectively analyzed, excluding those with revision and conversion surgeries, postoperative GLP-1 use, or pregnancy during follow-up. Propensity score matching (1:1) was used to adjust for confounders. The primary outcome was a combined endpoint of the SF-Bari Score, incorporating weight loss, improvement of obesity-related conditions, and surgical complications until 36 months post-surgery.

**Results:**

A total of 215 patients were analyzed, including 54 who received GLP-1 RAs prior to surgery, with a preoperative total body weight loss (%TWL) of 3.7 ± 4.3%. %TWL did not differ significantly between groups: at 12 months, 29.8 ± 7.7% (non-GLP-1) vs. 28.1 ± 7.6% (GLP-1); at 24 months, 28.9 ± 8.2% vs. 27.2 ± 8.4%; and at 36 months, 26.9 ± 9.1% vs. 25.4 ± 9.1%. SF-Bari Scores were similar between groups at all timepoints: 96.0 ± 24.2 vs. 93.5 ± 26.3 at 12 months, 93.8 ± 24.4 vs. 91.0 ± 25.6 at 24 months, and 89.1 ± 24.7 vs. 85.5 ± 25.8 at 36 months (non-GLP-1 vs. GLP-1). Propensity score matching confirmed comparable outcomes between groups. Patients transitioned to surgery for definitive treatment, side effects, or medication unavailability.

**Conclusion:**

Among patients who proceeded to surgery after GLP-1 therapy, prior GLP-1 use was not associated with different surgical outcomes.

**Supplementary Information:**

The online version contains supplementary material available at 10.1007/s11695-025-08136-5.

## Background

Obesity, a chronic and relapsing disease characterized by excess adipose tissue and associated with significant systemic dysfunction, remains a major global health challenge. The worldwide prevalence of obesity has more than tripled between 1975 and 2022, with 43% of adults aged 18 and older classified as overweight, and 16% living with obesity [1, 2].

This alarming trend has profound consequences, as obesity is closely linked to an increased risk of type 2 diabetes mellitus (T2D), cardiovascular disease, certain types of cancers, and early mortality [3, 4].

Addressing obesity requires a multifaceted approach that includes lifestyle management, pharmacotherapy, and surgical interventions. Lifestyle modifications, like dietary changes and exercise, are often the first-line strategy. However, long-term adherence to these changes is challenging, and the efficacy of lifestyle management alone is limited [5, 6]. For many patients, metabolic bariatric surgery (MBS) offers a more effective solution, with evidence supporting its superiority in achieving significant and sustained weight loss. Procedures such as sleeve gastrectomy (SG) and Roux-en-Y gastric bypass (RYGB), not only promote significant weight loss but also improve obesity-related conditions. Furthermore, MBS has demonstrated safety and efficacy across diverse populations and age groups [7, 8].

In recent years, pharmacotherapy, particularly glucagon-like peptide-1 receptor agonists (GLP-1 RAs), one class of Obesity Management Medications (OMMs), has emerged as a potential treatment option or adjunct to MBS [9]. Initially approved by the FDA in 2005 for the management of T2D, GLP-1 RAs were later approved for weight management, with semaglutide gaining approval in 2021 [10, 11]. These medications exert their effects by agonizing GLP-1 receptors to enhance insulin secretion, suppress glucagon release, delay gastric emptying, and promote satiety [9].

In addition to cost concerns [12, 13], recent trends in obesity treatment highlight a growing preference for OMMs over surgery [14]. The use of medications before and/or after MBS in a neoadjuvant and adjuvant manner, similar to cancer chemotherapy, has been proposed as an effective strategy for managing obesity and metabolic disease [15–19]. A recent study challenges this strategy by showing that neoadjuvant semaglutide did not provide benefits for weight loss, diabetes remission, or safety up to one year after surgery [20].

Despite the growing use of GLP-1 RAs for obesity management, especially at higher doses, the long-term effects of using these agents as neoadjuvant therapy before MBS remain unclear. Specifically, it is unknown whether preoperative GLP-1 RA treatment meaningfully influences postoperative outcomes such as weight loss and improvement in obesity-related conditions. This knowledge gap is increasingly relevant given the surge in GLP-1 RA prescriptions and ongoing discussions about how these medications compare to or complement surgical interventions.

This study aimed to assess whether using GLP-1 RAs before MBS affects weight loss and improvements in obesity-related conditions over three years, in patients who switched to surgery due to an suboptimal response to GLP-1 therapy. We also explored why these patients chose to move from GLP-1 treatment to surgery.

## Material and Methods

### Patients

In a retrospective examination, we analyzed all patients who underwent either Roux-en-Y gastric bypass (RYGB) or sleeve gastrectomy (SG) at the University Hospital Basel from January 2015 to December 2021. Informed consent was obtained from all participants as part of the hospital's mandatory quality control procedures. Ethical approval was obtained from the Ethics Commission of Northwest and Central Switzerland (BASEC ID 2018-02093).

Patients were excluded if they had undergone gastric banding as their primary procedure, revisional operations, received GLP-1 therapy before and after surgery, received GLP-1 therapy only after surgery, or experienced pregnancies during the follow-up period.

In accordance to Swiss guidelines [21], we included individuals with a Body Mass Index (BMI) ≥ 35 kg/m^2^ who had not responded to conservative treatment for two years. Once patients met the criteria for MBS, they underwent a comprehensive preoperative assessment journey including a multidisciplinary and interprofessional team. This extensive preoperative process included multiple appointments, educational sessions, additional laboratory and diagnostic testing, and individualized medical interventions tailored to the patient’s clinical need. GLP-1 RAs prescriptions began at our clinic in 2018 and were determined on a case-by-case basis at the discretion of the treating physician.

### Outcomes

Demographic data, early morbidity records, and follow-up details regarding weight loss, obesity-related conditions, and complications were retrospectively analyzed for all bariatric patients who underwent surgery at our institution.

The SF-Bari Score was used as a composite endpoint, assessing three critical components: total body weight loss (%TWL), calculated using following formula: [(weight at baseline-weight at follow-up)/weight at baseline]*100, improvement in obesity-related conditions (T2D, hypertension, dyslipidemia, obstructive sleep apnea syndrome (OSAS)), and the occurrence of surgical complications, measured using the Comprehensive Complication Index (CCI)) [22]. The SF-BARI score was recently validated in a large external cohort of 21,603 patients from the Dutch Audit for Treatment of Obesity (DATO) and the Scandinavian Obesity Surgery Registries (SOReg-Sweden and SOReg-Norway), confirming its applicability to real-world populations with minimal influence from baseline characteristic [23]. Outcomes were assessed up to 36 months.

### Statistical Analysis

Mean and standard deviation were used to summarize continuous data, while counts and percentages were used for categorical variables.

Continuous variables were assessed for normality using the Kolmogorov–Smirnov test (Supp. Table 1). Variables that followed a normal distribution (p > 0.05) were compared between groups using the independent samples t-test. Variables that did not meet the assumption of normality (p ≤ 0.05) were compared using the non-parametric Wilcoxon rank-sum test. Categorical variables were summarized as frequencies and percentages and compared using the chi-square test or Fisher’s exact test where appropriate. Differences in ordinal scaled data were assessed using Pearson's Chi-squared or Kruskal–Wallis test. McNemar's test was used to assess changes in diabetes status from start to stop of GLP-1 medication.

To minimize potential confounding and ensure a balanced comparison a propensity score matching analysis was performed. Using nearest-neighbor propensity score matching in a 1:1 ratio and a caliper of 0.2, we matched patients based on age, sex, preoperative BMI, hypertension, diabetes status, and surgical procedure. These variables were selected based on clinical relevance and availability. The model’s discriminative ability was evaluated by calculating the area under the receiver operating characteristic curve (AUC). Our approach aimed to reduce selection bias and improve the validity of our comparisons regarding postoperative outcomes. A p-value less than 0.05 was considered statistically significant. To assess whether the study was sufficiently powered to detect the observed difference in %TWL at 12 months, a post-hoc power analysis was conducted. The analysis was based on the observed unmatched group means and standard deviations. The standardized effect size (Cohen’s d) was calculated and used in a two-tailed independent-samples t-test power calculation (α = 0.05). Additionally, we estimated the required sample size to achieve 80% power to detect a difference of the observed magnitude under both balanced and unbalanced (3:1) group allocations using the pwr package in R. To assess the impact of missing data, a sensitivity analysis was performed, using multiple imputation by chained equations (MICE) with 5 imputed datasets. Predictive mean matching was used for continuous variables including %TWL at 12 months and SF-BARI score at 12 months. Linear regression models were run across each imputed dataset and pooled. All statistical analyses were performed using R 4.2.3 (R Foundation for Statistical Computing, Vienna, Austria).

## Results

### Study Population

A total of 215 patients were included for analysis. Among these, 161 patients had not received any GLP-1 RAs before or after surgery, while 54 patients had been treated with GLP-1 RAs prior to the scheduled MBS.

### Overall (unmatched population)

#### Baseline Characteristics

Baseline characteristics are shown in Table 1. There were no significant differences in age (43.0 ± 13.1 years versus 45.0 ± 12.2 years) or sex distribution (71.4% female versus 57.4% female) between patients treated with GLP-1 and those without GLP-1 prior to surgery. However, patients receiving GLP-1 therapy more frequently underwent RYGB compared to SG (72.2% versus 50.9%). Baseline BMI was similar between the two groups (41.1 ± 4.8 kg/m^2^ without GLP-1 and 42.3 ± 5.1 kg/m^2^ with GLP-1). A higher prevalence of T2D was observed in the GLP-1 group (37.0% versus 11.8%), accompanied by a higher HbA1c (6.2 ± 1.0% versus 5.6 ± 0.7%). Hypertension was also more common in the GLP-1 group (59.6% versus 40.4%). No significant differences were found in the prevalence of hyperlipidemia or OSAS) between the two groups.

**Table 1:** Baseline-characteristics (unmatched groups)

Variable	No GLP-1 (n = 161)	GLP-1 (n = 54)	p-value
**Age (years)**	43.0 ± 13.1	45.0 ± 12.2	0.444
**Sex**			0.082
Female	115 (71.4%)	31 (57.4%)	
Male	46 (28.6%)	23 (42.6%)	
**Defining Surgery**			**0.010**
SG	79 (49.1%)	15 (27.8%)	
RYGB	82 (50.9%)	39 (72.2%)	
**BMI (kg/m**^**2**^)*	41.1 ± 4.8	42.3 ± 5.1	0.126
**T2D**	19 (11.8%)	20 (37.0%)	** < 0.001**
**HbA1c (%)**	5.6 ± 0.7	6.2 ± 1.0	**0.001**
**Hypertension**	65 (40.4%)	31 (59.6%)	**0.048**
**Hyperlipidemia**	53 (32.9%)	26 (49.1%)	0.097
**OSAS**	55 (34.2%)	15 (28.3%)	0.607

*SG* sleeve gastrectomy, * RYGB* Roux-en-Y gastric bypass,* BMI* Body mass index,* T2D* type 2 diabetes mellitus,* OSAS* obstructive sleep apnea syndrome

Data show mean and standard deviation, unless otherwise specified

*at time of surgery

Patients treated with GLP-1 RAs received treatment for an average of 9.2 ± 8.6 months (Table 2). Of those, 21 (38.8%) were treated with semaglutide, 30 (55.6%) with liraglutide, and 3 (5.6%) with dulaglutide. There was no significant difference in BMI between the start (43.2 ± 5.9 kg/m^2^) and stop (41.3 ± 5.2 kg/m^2^) of GLP-1 therapy. Additionally, %TWL was similar across all three medications. Although HbA1c showed a statistically significant reduction from start (6.3 ± 1.2%) to stop (6.0 ± 0.9%, p = 0.017) of medication, T2D rates for T2D did not significantly differ (48.1% at baseline vs. 40.7% at follow-up, p = 0.125) with a remission rate of 15.4% (Table 2)*.*

**Table 2:** Characteristics of patients with GLP-1-agonists

Variable	GLP-1 (n = 54)	p-value
**GLP-1 medication**		
Semaglutide	21 (38.8%)	
Liraglutide	30 (55.6%)	
Dulaglutide	3 (5.6%)	
**Duration of GLP-1 therapy (months)**	9.2 ± 8.6	
**Side Effects**		
Gastrointestinal	14 (25.9%)	
Allergic reaction	1 (1.9%)	
Dizziness	1 (1.9%)	
Depression	1 (1.9%)	
**Reasons for discontinuation of GLP-1**		
Insufficient clinical response	17 (31.5%)	
Side effects	12 (22.2%)	
Availability	5 (9.3%)	
Patient wanted “definite” treatment	20 (37.0%)	
**BMI (kg/m**^**2**^**)**		0.071
at start of GLP-1	43.2 ± 5.9	
at stop of GLP-1	41.3 ± 5.2	
TWL (%)*		0.883
Overall	3.7 ± 4.3	
Semaglutide	3.8 ± 4.0	
Liraglutide	3.8 ± 4.7	
Dulaglutide	2.7 ± 2.4	
**HbA1c (%)**		**0.017**
at start of GLP-1	6.3 ± 1.2	
at stop of GLP-1	6.0 ± 0.9	
**T2D**		0.125
at start of GLP-1	26 (48.1%)	
at start of GLP-1	22 (40.7%)	

*BMI* Body mass index*, TWL* total body weight loss,* T2D* type 2 diabetes mellitus

Data show mean and standard deviation, unless otherwise specified

*between start – stop

#### Reasons for Choosing Surgery after GLP-1 Treatment

Among patients in the GLP-1 group, the majority (n = 20; 37.0%) opted for surgery due to a desire for a definitive treatment (Table 2). Twelve patients (22.2%) discontinued GLP-1 treatment due to side effects, which included gastrointestinal symptoms (n = 14), dizziness (n = 1), and depression (n = 1) and an allergic reaction in one patient. Overall, 17 patients (31.5%) experienced side effects, although two patients with gastrointestinal symptoms were able to continue treatment. Additionally, 5 patients (9.3%) discontinued GLP-1 therapy due to product unavailability.

#### Weight Loss

%TWL between the start and stop of preoperative GLP-1 treatment was 3.7 ± 4.3% (Table 2).

Individual %TWL for semaglutide, liraglutide, and dulaglutide was 3.8 ± 4.0%, 3.8 ± 4.7% and 2.7 ± 2.4%, respectively. Follow-up for the %TWL between start and stop of GLP-1 RA was 96.3%.

After surgery, %TWL was comparable between the two groups across all three years of follow-up (Table 3). After one year, patients without GLP-1 treatment lost 29.8 ± 7.7% of their body weight, compared to 28.1 ± 7.6% in those with GLP-1. After two years, the weight loss was 28.9 ± 8.2% for the non-GLP-1 group and 27.2 ± 8.4% for the GLP-1 group. At the three-year mark, patients without GLP-1 had a weight loss of 26.9 ± 9.1%, while those with GLP-1 lost 25.4 ± 9.1% of their total body weight.

**Table 3:** Outcomes (unmatched groups)

Variable	No GLP-1 (n = 161)	GLP-1 (n = 54)	p-value
**BMI (kg/m**^**2**^**)**			
12 months	28.9 ± 4.7	30.5 ± 5.2	**0.047**
24 months	29.4 ± 5.0	30.3 ± 6.1	0.063
36 months	30.1 ± 5.3	31.5 ± 5.6	0.147
**T2D**			
12 months	3 (1.8%)	3 (5.5%)	0.366
24 months	4 (2.5%)	3 (5.5%)	0.127
36 months	2 (1.2%)	2 (3.7%)	0.458
**HbA1c (%)**			
12 months	5.2 ± 0.8	5.3 ± 0.3	0.201
24 months	5.2 ± 0.8	5.2 ± 0.4	0.067
36 months	5.3 ± 0.6	5.5 ± 0.9	0.601
**Hypertension**			
12 months	33 (23.9%)	18 (36.7%)	0.122
24 months	28 (23.0%)	16 (39.0%)	0.071
36 months	25 (24.0%)	15 (50.0%)	**0.012**
**Hyperlipidemia**			
12 months	29 (21.0%)	12 (24.5%)	0.761
24 months	25 (20.5%)	11 (26.8%)	0.530
36 months	22 (21.2%)	11 (35.5%)	**0.042**
**OSAS**			
12 months	20 (14.6%)	6 (12.0%)	0.232
24 months	13 (10.7%)	6 (14.6%)	0.685
36 months	11 (10.6%)	4 (13.3%)	0.926
**TWL (%)**			
12 months	29.8 ± 7.7	28.1 ± 7.6	0.177
24 months	28.9 ± 8.2	27.2 ± 8.4	0.213
36 months	26.9 ± 9.1	25.4 ± 9.1	0.296
**SF-BARI Score**			
12 months	96.0 ± 24.2	93.5 ± 26.3	0.657
24 months	93.8 ± 24.4	91.0 ± 25.6	0.589
36 months	89.1 ± 24.7	85.5 ± 25.8	0.490
**CCI**	2.59 ± 8.3	3.0 ± 8.6	0.879

*BMI* Body mass index,* T2D* type 2 diabetes mellitus,* OSAS* obstructive sleep apnea syndrome,* TWL* total body weight loss,* CCI* Comprehensive Complication Index

Data show mean and standard deviation, unless otherwise specified

Follow-up rates for the postoperative %TWL were 85.1% for 12 months,74.0% after 24 months, 62.3% after 36 months.

#### Obesity-Related Conditions

The prevalence of T2D and OSAS was similar between the two groups at all time points, with no significant difference in HbA1c levels (Table 3). While hypertension and dyslipidemia rates were comparable at 12 and 24 months, at 36 months, patients who had received preoperative GLP-1 RAs had higher rates of hypertension (50.0% vs. 24.0%) and dyslipidemia (35.3% vs. 21.2%).

The SF-Bari Score, used as a composite endpoint, showed similar outcomes in both groups (Table 3). A"good response"was achieved for both groups at all time points, with scores of 96.0 ± 24.2 in the non-GLP-1 group and 93.5 ± 26.3 in the GLP-1 group at 12 months, 93.8 ± 24.4 versus 91.0 ± 25.6 at 24 months, and 89.1 ± 24.7 versus 85.5 ± 25.8 at 36 months. Follow-up rates for the SF-Bari Score were 85.1% at 12 months, 73.5% at 24 months, and 65.1% at 36 months.

#### Complications

Detailed information on complications is presented in Table 4. Overall, the incidence of complications within the first 36 months after surgery was similar between the groups, occurring in 23 patients (14.3%) in the group without prior GLP-1 treatment and 8 patients (14.8%) in the GLP-1 group. Among patients without GLP-1 treatment, the most common complications were hematoma (n = 5; 3.1%), postoperative intestinal dysmotility (n = 4; 2.5%) and wound infections (n = 4; 2.5%). Hematoma was also the most frequent complication in the GLP-1 group (n = 4; 7.4%). The severity of complications, measured using the CCI, was also comparable between the groups (2.6 ± 8.3 vs. 3.0 ± 8.6).

**Table 4:** Overall complications at 36 months between groups (unmatched)

	No GLP-1 (n = 161)	GLP-1 (n = 54)	p-value
Overall (n)	23 (14.3%)	8 (14.8%)	1.000
Urinary retention	2	2	
Intestinal dysmotility	4	1	
Intestinal obstruction	1	1	
Wound infection	4	2	
Hematoma	5	4	
Pancreatitis	1	0	
Stenosis of gastrojejunostomy	1	0	
Asystole	1	0	
Perforation	1	0	
Gastrointestinal bleeding	2	0	
Pulmonary embolism	1	0	
CCI	2.6 ± 8.3	3.0 ± 8.6	0.879

*CCI* Comprehensive Complication Index

Data show mean and standard deviation, unless otherwise specified

## Post-hoc Power Analysis

The observed mean difference in %TWL between both groups was 1.7 percentage points, cor- responding to a small effect size (Cohen’s d = 0.22). The post-hoc power analysis indicated that the study had approximately 29% power to detect this difference at a significance level of 0.05.

## Sensitivity Analysis

In the complete-case analysis (n = 160), GLP-1 therapy was associated with a non-significant increase in %TWL at 12 months (mean difference = 1.17%, 95% CI: [–4.53, 6.87], p = 0.688) and a non-significant increase in SF-BARI score at 12 months (mean difference = 1.31 points, 95% CI: [–6.41, 9.03], p = 0.771). The multiple imputation analysis yielded similar results, with no statistically significant association between GLP-1 therapy and either outcome. The consistency across complete-case and imputed analyses supports the robustness of our findings.

### Matched Population

To reduce potential confounding factors and achieve a more balanced comparison between patients, a 1:1 ratio propensity score matching was conducted. Two cohorts, each consisting of 54 patients, were formed. The propensity score model showed fair ability to distinguish between treated and control patients, with an AUC of 0.717, supporting its use for estimating the probability of treatment assignment. Detailed balance statistics illustrating covariate balance before and after matching are presented in the supplementary materials (Supp. Table 2).

### Baseline Characteristics

Details are presented in Table 5*.* Baseline characteristics were comparable between the cohorts in terms of age, sex, type of surgery, BMI at the time of surgery, and obesity-related conditions.

**Table 5:** Baseline-characteristics (matched groups)

Variable	No GLP-1 (n = 54)	GLP-1 (n = 54)	p-value
**Age (years)**	43.7 ± 14.0	44.4 ± 12.2	0.793
**Sex**			0.846
Female	29 (53.7%)	31 (57.4%)	
Male	25 (46.3%)	23 (42.6%)	
**Defining Surgery**			
SG	18 (33.3%)	15 (27.8%)	0.676
RYGB	36 (66.7%)	39 (72.2%)	
**BMI (kg/m**^**2**^)*	41.2 (± 4.6)	42.3 (± 5.1)	0.271
**T2D**	17 (31.5%)	20 (37.0%)	0.685
**HbA1c (%)**	5.8 ± 0.8	6.2 ± 1.0	0.089
**Hypertension**	23 (42.6%)	31 (59.6%)	0.154
**Hyperlipidemia**	18 (33.3%)	26 (49.1%)	0.176
**OSAS**	19 (35.2%)	15 (28.3%)	0.431

*SG* sleeve gastrectomy,* RYGB* Roux-en-Y gastric bypass,* BMI* Body mass index,* T2D* type 2 diabetes mellitus,* OSAS* obstructive sleep apnea syndrome

Data show mean and standard deviation, unless otherwise specified

*at time of surgery

### Weight Loss

**Fig. 1:**
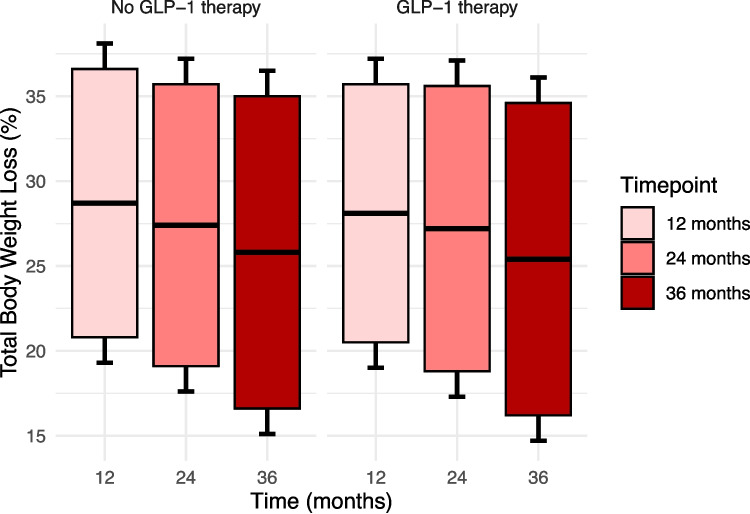
Total body weight loss (%TWL) between matched groups. Boxplots showing the mean (central line) and standard deviation (whiskers)

There was no significant difference in %TWL between the matched groups at any time point (Table 6) (Fig. 1). At 12 months, %TWL was 28.7 ± 7.9% in the non-GLP-1 group and 28.1 ± 7.6% in the GLP-1 group. Similarly, at 24 months, %TWL was 27.4 ± 8.3% in the non-GLP-1 group and 27.2 ± 8.4% in the GLP-1 group, and at 36 months, %TWL was 25.8 ± 9.2% and 25.4 ± 9.2%, respectively.

**Table 6:** Outcomes (matched groups)

Variable	No GLP-1 (n = 54)	GLP-1 (n = 54)	p-value
**BMI**			
12 months	29.3 ± 4.4	30.5 ± 5.2	0.248
24 months	29.9 ± 5.0	53.9 ± 149.5	0.314
36 months	30.6 ± 5.9	31.5 ± 5.6	0.556
**T2D**			
12 months	2 (4.0%)	3 (6.1%)	0.982
24 months	3 (7.5%)	3 (7.3%)	0.610
36 months	2 (6.5%)	2 (6.5%)	1.000
**HbA1c (%)**			
12 months	5.4 ± 1.0	5.3 ± 0.3	0.352
24 months	5.4 ± 0.9	5.3 ± 0.3	0.311
36 months	5.6 ± 1.0	5.5 ± 0.9	0.719
**Hypertension**			
12 months	13 (26.0%)	18 (36.7%)	0.350
24 months	12 (29.3%)	16 (39.0%)	0.485
36 months	9 (30.0%)	15 (50.0%)	0.188
**Hyperlipidemia**			
12 months	12 (24.0%)	12 (24.5%)	1.000
24 months	10 (24.4%)	11 (26.8%)	1.000
36 months	5 (16.7%)	11 (35.5%)	0.132
**OSAS**			
12 months	7 (14.3%)	6 (12.0%)	0.583
24 months	4 (9.8%)	6 (14.6%)	0.736
36 months	3 (10.0%)	4 (13.3%)	1.000
**TWL (%)**			
12 months	28.7 ± 7.9	28.1 ± 7.6	0.837
24 months	27.4 ± 8.3	27.2 ± 8.4	0.973
36 months	25.8 ± 9.2	25.4 ± 9.2	0.882
**SF-BARI-Score**			
12 months	94.7 ± 24.9	93.5 ± 26.3	0.947
24 months	89.5 ± 24.5	91.0 ± 25.6	0.596
36 months	85.6 ± 24.9	85.5 ± 25.8	0.808
**CCI**	3.2 ± 9.4	3.0 ± 8.6	0.930

*BMI* Body mass index,* T2D* type 2 diabetes mellitus,* OSAS* obstructive sleep apnea syndrome,* TWL* total body weight loss,* CCI* Comprehensive Complication Index

Data show mean and standard deviation, unless otherwise specified

### Obesity-Related Conditions

**Fig. 2:**
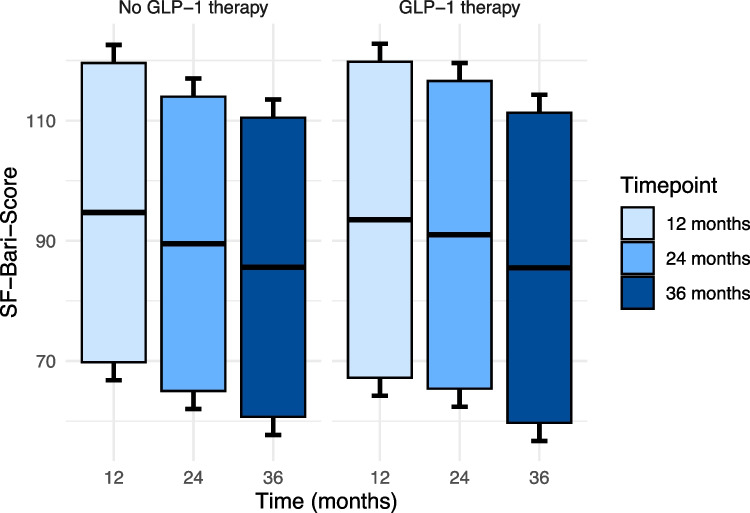
SF-Bari score between matched groups. Boxplots showing the mean (central line) and standard deviation (whiskers)

The prevalence of T2D, hypertension, hyperlipidemia, and OSAS was comparable between the groups at all time points (Table 6)*.* Similarly, resolution of obesity-related conditions, as assessed by the SF-Bari Score, showed similar values for both groups: 94.7 ± 24.9 for patients without prior GLP-1 treatment versus 93.5 ± 26.3 for those with prior GLP-1 treatment at 12 months, 89.5 ± 24.5 versus 91.0 ± 25.6 at 24 months, and 85.6 ± 24.9 versus 85.5 ± 25.8 at 36 months (Table 6) (Fig. 2). Both groups were again classified as having a"good response"at all time points. At 12, 24, and 36 months, the T2D remission rates were 88.2%, 82.4%, and 88.2% in the No-GLP-1 group, and 85.0%, 85.0%, and 90.0% in the GLP-1 group, respectively.

### Complications

The CCI showed no differences between the groups, with a score of 3.2 ± 9.4 in the non-GLP-1 group and 3.0 ± 8.6 in the GLP-1 group.

## Discussion

The objectives of the current study were to evaluate differences in weight loss and improvement of obesity-related conditions between MBS patients with and without prior GLP-1 RA treatment up to 36 months after surgery, as well as to explore reasons for transitioning from GLP-1 therapy to surgery. To our knowledge, this study provides the longest follow-up to date on the outcomes of preoperative GLP-1 RA use in MBS patients. Our findings suggest that preoperative GLP-1 treatment was not associated with impoved outcomes, as assessed by the SF-Bari Score as a composite endpoint. Additionally, there was no difference in the incidence and severity of postoperative complications. Our findings did not change after matching for age, sex, preoperative BMI, diabetes status, and surgical procedure. Most patients in the GLP-1 group pursued surgery for definitive treatment or due to limitations in GLP-1 therapy, such as side effects or product unavailability.

Our findings align with a recently published study that also used nearest-neighbor propensity score matching to compare 182 patients who received preoperative semaglutide before MBS with 182 controls [20]. The study followed patients for up to 12 months and found that neoadjuvant semaglutide did not result in significant benefits for weight loss, diabetes remission, or surgical safety.

Weight loss following a mean preoperative GLP-1 RA treatment duration of 9.2 months was modest, with a mean reduction of only 3.7 ± 4.3%, and no significant differences observed between semaglutide and liraglutide. In contrast, weight loss at 12 months post-surgery was substantially greater, with reductions of 29.8 ± 7.7% and 28.1 ± 7.6%, respectively. In our study, postoperative weight loss was equal between patients with and without prior GLP-1 RA treatment at all time points. This contrasts with a study by Ilange et al., which suggests that GLP-1 agonist use prior to MBS in patients with a BMI > 50 kg/m^2^ results in significantly greater weight loss prior to MBS, without delaying surgery or increasing complication rates [15]. They concluded that these medications may enhance perioperative safety and improve long-term outcomes in this very high-risk BMI population. Similarly, a propensity score matched study including 13,129 patients and 35,020 procedures showed that the perioperative use of GLP-1 RA in patients with diabetes was associated with a significant reductions in risk-adjusted readmission, wound dehiscence, and occurrence of hematoma [24].

In our study, the prevalence of diabetes at the time of surgery was higher in the GLP-1 group (37.0% vs. 11.8%). This can be explained by the fact that GLP-1 RAs were preferentially prescribed to patients with T2D. Several randomized trials have investigated the safety and efficacy of GLP-1 RAs in patients with T2D who either had known cardiovascular disease or were at high risk for it [25–28]. These studies used a composite of major adverse cardiovascular events (myocardial infarction, stroke, or death from cardiovascular causes) as the primary endpoint and have demonstrated favorable results.

Another potential application of preoperative GLP-1 therapy could be as a bridge to surgery in patients with severe obesity who are initially deemed inoperable. Stier et al. demonstrated that a short-term regimen of liraglutide, combined with a leucine-rich amino acid infusion and a hypocaloric diet, enabled technical operability within 2–4 weeks in 26 patients with acutely life-threatening obesity who were not considered fit for surgery after anesthesiology consultation [29].

In the face of the growing prescription rates and popularity of GLP-1 analogs, it remains unknown what the trend for MBS will be in the coming years. A recent cross-sectional study from the US reported a more than two-fold increase in the use of GLP-1 RAs as anti-obesity medications from 2022 to 2023, accompanied by a 25.6% decline in MBS rates during the same period [14]. While GLP-1 analogs are effective for managing obesity and related conditions such as diabetes, their high cost and gastrointestinal side effects often result in treatment discontinuation and subsequent recurrent weight gain [30]. This was also reflected in our study with 25.6% experiencing adverse effect and 23.3% having to discontinue treatment due to these side effects.

A recently published analysis by Docimo et al. highlights the cost implications of GLP-1 therapy versus MBS [12]. Although GLP-1 RAs appear less expensive initially, their costs quickly surpass those of MBS within 1 to 1.5 years of treatment, depending on the specific medication and procedure. The delayed effectiveness of GLP-1 RAs and the potential for recurrent weight gain upon discontinuation further diminish their cost-effectiveness in the long term. Results from the STEP 1 trial extension showed that one year after withdrawal of once-weekly subcutaneous semaglutide 2.4 mg and lifestyle intervention, participants experienced recurrent weight gain of two-thirds of their prior weight loss [31]. In contrast, MBS provides more immediate and sustained weight loss, addresses obesity-related conditions more effectively, and, despite higher upfront costs, offers greater value to both the healthcare system and the patient over time [32].

An intriguing and potentially more impactful application of GLP-1 RAs may lie in their postoperative use, particularly in patients who experience suboptimal clinical outcomes following MBS. Emerging evidence suggests that GLP-1 RA therapy can significantly enhance weight loss outcomes in such cases [33].

For example, a retrospective observational study by Jensen et al. explored the efficacy of liraglutide and semaglutide in patients who regained 15.1% of their total body weight after MBS [19]. Their findings revealed that six months of GLP-1 RA therapy led to an 8.8% reduction in total weight and a decrease of 2.9 kg/m^2^ in BMI. Remarkably, patients undergoing GLP-1 RA treatment were able to lose on average two-thirds of the weight regained from their nadir weight. Other studies have reported an additional weight reduction of 5.3%–10.3% within 3–9 months with postoperative GLP-1 treatment [34–37].

What remains unknown is the association between the response to GLP-1 RAs and subsequent MBS outcomes [38]. Future studies should focus on subgroup analyses to compare postoperative outcomes between patients who initially responded well to GLP-1 therapy and those who experienced suboptimal or no weight loss. Such research would provide valuable insights into the effectiveness of GLP-1 analogs in optimizing bariatric outcomes and help identify which patients may benefit most from preoperative GLP-1 treatment.

Some limitations of this study should be considered. First, the retrospective, single center-design may affect generalizability of the findings and warrants external validation. Second, the observational design of this study may introduce selection bias. Specifically, patients who ultimately underwent MBS after GLP-1 therapy may differ systematically—such as in motivation or treatment response—from those who did not proceed to surgery. This study exclusively included patients with suboptimal response to GLP-1 therapy who subsequently underwent surgery. Patients who achieved adequate weight loss with GLP-1 therapy alone were not included, potentially overrepresenting non-responders and limiting causal inference. Therefore, these findings cannot inform broader clinical decisions comparing GLP-1 therapy effectiveness to surgical interventions and apply only to the subset of patients who are candidates for surgery after suboptimal response to medical management.

However, it is important to note that patients with suboptimal weight loss after OMM treatment are the ones most referred to our outpatient clinic. Consequently, these patients represent a critical subset for bariatric surgeons, as they are the most relevant candidates for surgical intervention. While this study represents the longest report of preoperative GLP-1 use to date, the 36-month follow-up limits assessment of long-term durability. Additionally, we experienced a significant loss to follow-up and a relatively small sample size, which may have influenced the reliability of our results. This further limited our ability to perform subgroup analyses with implications for clinical interpretation. These findings warrant validation in larger, randomized controlled trials. Finally, even though our propensity matching resulted in fair discriminatory ability, residual confounding cannot be entirely excluded.

In our study, only single-receptor GLP-1 agonists were used. However, dual-receptor agonists like tirzepatide, and triple-receptor agonists such as retatrutide, have shown greater weight loss potential [39, 40]. Applying these medications prior to surgery could potentially lead to better outcomes, enhancing the effects of weight loss and improving the outcomes of bariatric procedures.

The main strength of our study lies in its reflection of real-world clinical practice derived from a high-volume bariatric center, as it includes not only high-risk patients but the full spectrum of individuals undergoing MBS in our daily clinic. By capturing a diverse patient population, our findings provide a comprehensive and applicable perspective on the impact of preoperative GLP-1 therapy in routine surgical care. We strengthened our findings by performing propensity score matching to balance the groups, as patients in the GLP-1 cohort were metabolically more ill at baseline, with a higher prevalence of T2D and elevated HbA1c levels in the unmatched cohort. This approach enhances the validity of our results by minimizing potential confounding factors.

## Conclusion

In conclusion, while GLP-1 RAs and MBS both address obesity and its associated medical problems, their applications and outcomes differ. Our findings suggest that preoperative GLP-1 therapy was not associated with improved surgical outcomes but probably delays the more effective surgical treatment. As trends in obesity treatment continue to evolve, further research is essential to define the optimal integration of GLP-1 therapy and MBS to maximize patient benefits.

## Supplementary Information

Below is the link to the electronic supplementary material.

Supplementary file1 (DOCX 18 KB)

## Data Availability

The data and codes relating to the study are available from the corresponding author upon reasonable request.
