# Supplementary material for: Surgical Outcomes in Patients with Preoperative GLP-1 Therapy: A Retrospective Analysis
Source: Obes Surg. 2025 Aug 12;35(9):3847–57. doi: 10.1007/s11695-025-08136-5 (PMC12457577; doi:10.1007/s11695-025-08136-5)
Supplement: Supplementary file 1 — Supplementary file1 (DOCX 18 KB) [file 11695_2025_8136_MOESM1_ESM.docx]

Supplementary Table 1: Summary of normality assessment and selected statistical tests

| **Variable** | **p-value** | **Normality** | **Statistical test** |
| --- | --- | --- | --- |
| Age | 0.51 | Yes | t-test |
| HbA1c_baseline | <0.001 | No | Wilcoxon rank-sum test |
| HbA1c_1 year | <0.001 | No | Wilcoxon rank-sum test |
| HbA1c_2 years | <0.001 | No | Wilcoxon rank-sum test |
| HbA1c_3 years | <0.001 | No | Wilcoxon rank-sum test |
| BMI_baseline | 0.32 | Yes | t-test |
| BMI_1 year | 0.35 | Yes | t-test |
| BMI_2 years | <0.01 | No | Wilcoxon rank-sum test |
| BMI_3 years | 0.14 | Yes | t-test |
| %TWL_1 year | 0.78 | Yes | t-test |
| %TWL_2 years | 0.82 | Yes | t-test |
| %TWL_3 years | 0.70 | Yes | t-test |
| SF-Bari_1 year | 0.57 | Yes | t-test |
| SF-Bari_2 years | 0.81 | Yes | t-test |
| SF-Bari_3 years | 0.78 | Yes | t-test |

*BMI=Body mass index; %TWL=total body weight loss*

Supplementary Table 2: Differences between both groups before and after matching

| **Balance Measures** | **Diff. Unadjusted** | **Diff. Adjusted** | **Balance** | **Threshold** |
| --- | --- | --- | --- | --- |
| Age | 0.72 | 0.02 | Balanced | <0.1 |
| Sex | 0.12 | -0.05 | Balanced | <0.1 |
| BMI at baseline | 0.14 | 0.09 | Balanced | <0.1 |
| Diabetes at baseline | 0.23 | 0.07 | Balanced | <0.1 |
| Hypertension at baseline | 0.17 | 0.01 | Balanced | <0.1 |
| Defining Surgery | 0.21 | 0.09 | Balanced | <0.1 |
